# Predator loss amplifies the disruptive impacts of heatwaves

**DOI:** 10.1098/rspb.2026.2081

**Published:** 2026-11-04

**Authors:** Daniel Gibson, Adam Siepielski, Samuel B. Fey

**Affiliations:** ^1^ Department of Biology, Reed College, Portland, OR, USA; ^2^ Department of Biological Sciences, University of Arkansas, Fayetteville, AR, USA

**Keywords:** catastrophes, defaunation, rare demographic events, thermal stress

## Abstract

Heatwaves are intensifying under climate change, often triggering the catastrophic loss of predators. Still, it remains unclear whether their ecological impacts on ecosystems arise from direct thermal stress or from altered food web dynamics following predator loss. Using experimental tri-trophic fresh water food webs, we independently manipulated heatwave intensity (none, moderate or severe) and predator persistence (fish removed during the onset of heatwaves versus fish persisted during heatwaves) to disentangle these pathways. We show that predator loss, not heat alone, is the primary driver of community restructuring following a heatwave. Predator loss produced strong trophic cascades, and communities became dominated by large Daphniidae consumers that were less diverse, less temporally stable and more synchronized. These changes coincided with declines in primary producers despite increased nutrient availability from decomposing fish. By contrast, direct thermal effects were modest and depended strongly on trophic structure. When predators persisted, heatwaves intensified top-down pressure, producing declines in predator-sensitive consumers. When predators were lost, heatwaves instead magnified trophic disruptions by releasing vulnerable consumers from top-down control, generating substantial and persistent shifts in community structure. Ecosystems that retain predators may therefore be more resilient to heatwaves, underscoring predator conservation as a key strategy for maintaining ecosystem stability under accelerating climate extremes.

## 1. Introduction

Understanding how extreme temperatures shape ecological processes is central for accurately anticipating the effects of ongoing global environmental change [1,2]. Heatwaves—periods of abnormally high temperatures—are becoming more frequent, intense and prolonged [3,4]. These events can have staggering physiological and ecological effects, triggering mass mortality events across taxa [5,6], restructuring food webs [7–9] and initiating long-term shifts in biodiversity [10]. However, because such shifts in community structure and the direct physiological impacts of extreme temperatures on community members occur nearly simultaneously, their relative ecological importance during and after heatwaves remains unclear.

One of the most consequential impacts of heatwaves may be the loss of predators [11,12], which have key roles in shaping communities and ecosystems [13–16] and appear disproportionally sensitive to thermal extremes [17–19]. Such predator losses can lead to sustained trophic downgrading by weakening top-down control and altering nutrient dynamics that limit primary productivity [9,12,20,21]. For example, the recent die-off of 4 million common murres (*Uria aalge*), a key predator in marine ecosystems, during a heatwave in Alaska may have led to a pronounced ecosystem shift [22] highlighting both the thermal vulnerability of predators and the relationship between altered food web dynamics and predator loss. Although heatwaves are known to directly stress both primary producers and consumers [5] and can also trigger predator die-offs [5,6], it remains unclear whether direct thermal stress or the loss of predators contributes more strongly to food web restructuring.

In addition to extirpating predators, temperature also shapes biological rates [23], community size structure [24,25] and consumer-resource dynamics [26–28]. Elevated temperatures are expected to strengthen top-down control because consumers’ *per capita* metabolic demand increases [29,30]. However, these predictions may fail at extreme temperatures when feeding rates decline [31,32], resource availability is reduced or functional responses shift [33]. As a result, heatwaves may generate nonlinear and unpredictable community responses, with empirical studies reporting both destabilization [33] and enhanced stability of communities [34]. Moreover, it is unclear whether predator loss will magnify or obscure the direct thermal effects of heatwaves. Previous studies have shown strengthened top-down control during periods of warming [33,35,36], whereas others report minimal effects [37] or even weakened predation [38,39]. The impact of both heatwaves and fish die-offs may additionally impact community synchrony according to their intensity. For example, low severity heatwaves may reduce community synchrony by decreasing the abundance of temperature-sensitive taxa while benefiting taxa tolerant of higher temperature [40], while extremely high severity heatwaves may increase community synchrony as such outcomes are associated with coordinated declines in the abundance of all taxa [41].

Despite clear evidence linking heatwaves to predator declines and food web disruption, previous studies cannot resolve their relative importance for several reasons. Although observational accounts of heatwaves reveal the capacity for heatwaves to produce rapid ecological change [42], they do not facilitate mechanistic, causal insight into such outcomes. Warming experiments, meanwhile, have primarily used a constant elevated mean temperature rather than acute heatwaves of different intensities [43] and have employed predator exclusions rather than simulating natural mortality, where decomposing animal carrion generates an important fertilization effect that can increase primary production [9,44]. Existing experiments have also focused on changes in biomass or interaction strength, rather than how community composition, stability and post-heatwave transient dynamics unfold. Understanding these latter effects is necessary to better understand community resilience and stability—key underpinnings of ecosystem properties—to heatwaves.

Here, we use an experimental approach in a tri-trophic fresh water food web, a model system with strong consumer-resource linkages that is also facing unprecedented warming [4,45], to implement heatwave intensity treatments to disentangle the direct effects of temperature (producing either no heatwave, a moderate heatwave or a severe heatwave) from the indirect trophic-mediated effects of predator loss (either removing fish during the onset of heatwave or allowing fish to remain and survive the heatwave). By imposing heatwave treatments while simultaneously simulating predator mortality through carcass additions, this design seeks to capture the transient ecological dynamics that occur during heatwave-induced die-off events. We ask the following. (i) Are community responses to heatwaves driven primarily by direct thermal stress or by the disruption of predator-driven trophic cascades? (ii) Do producers and consumers experience greater effects of heatwaves when predators persist or when they perish? (iii) Do the effects of heatwave severity result in linear or nonlinear responses for consumers and producers? Our results reveal how biodiversity loss stemming from predator mortality and climate extremes interact to determine ecosystem vulnerability and identify predator persistence as a key determinant of ecological resilience in an increasingly volatile world.

## 2. Material and methods

We conducted a mesocosm experiment to investigate the independent and combined effects of heatwaves and predator die-offs in tri-trophic fresh water food webs containing phytoplankton primary producers, zooplankton primary consumers and fish predators. To do so, we employed a fully factorial, 3 × 2 experiment using an array of programmable heated mesocosms [46]. Each mesocosm was assigned to one of three levels of heatwave (no heatwave, moderate heatwave or severe heatwave) and two levels of predator loss (P−, in which all predators were removed, euthanized and replaced at the beginning of the heatwave; or P+, in which predators persisted throughout the experiment). This approach of replacing dead fish allows for predator decomposition and nutrient release to occur as happens in nature [9,47]. While heatwaves can result in the complete extirpation of fish within lakes [6,48], in many systems heatwave-induced fish mortality may be partial such that surviving individuals can still exert top-down control [48]. Thus, we use this extreme outcome to represent heatwaves that lead to complete local predator extirpation events. Four replicate mesocosms were used for each treatment, for a total of 24 mesocosms.

We chose to conduct this experiment using a mesocosm approach within a tri-trophic fresh water food web for several reasons. First, fresh water mesocosms have long been used to approximate the ecological processes of lake ecosystems on an experimentally feasible scale [49] that permits controlled and replicated experiments [9,49]. Second, the tri-trophic food web of phytoplankton, zooplankton and zooplanktivorous fish represents the dominant component of many fresh water lake and pond communities. These communities contain strong trophic linkages, such as between phytoplankton and herbivorous zooplankton [50] and between zooplankton and zooplanktivorous fish [51], which are often the top predators in ponds [52]. Third, heatwaves occur regularly in lake and pond ecosystems, are projected to become more frequent and intense over the 21st century [4] and often lead to die-offs of predatory fish [48].

### (a) Mesocosm set-up and community establishment

We arranged 24 cylindrical, low-density polyethylene containers (49 cm diameter × 58 cm height, 75.5 l volume) in two rows of 12 within an indoor, temperature-controlled greenhouse at Reed College, Oregon, USA, which typically maintained mesocosm water temperatures from 20.5°C to 23.2°C (supplementary material, figure S1). This size mesocosm was chosen for its ability to replicate the shallow depth of many ponds in hummocks of the Cascade Mountains near Mount St Helens (figure 1a) and because this array allowed a precise regulation of water temperature [46]. Conducting the experiment within a greenhouse, rather than in an outdoor mesocosm array, necessarily reduced exposure to natural variation in weather, light and other environmental drivers. While the greenhouse environment represents a simplification of natural conditions, it allowed us to isolate the direct and indirect effects of extreme warming with a level of control and replication that would not be feasible in the field. Although all mesocosms were lit from above (see §2b), because the room was exposed to sunlight through windows on the ceiling and south wall, we blocked the heatwave and predator loss treatments by row after a Welch's *t*-test revealed a difference in irradiance (measured in PPFD) between the rows (*t*_12.7_ = 4.18, *p* = 0.001), and row was added as a blocking factor in statistical models (see §2d). Mesocosms were randomly assigned to treatments within each row (supplementary material, figure S2).

**Figure 1.**
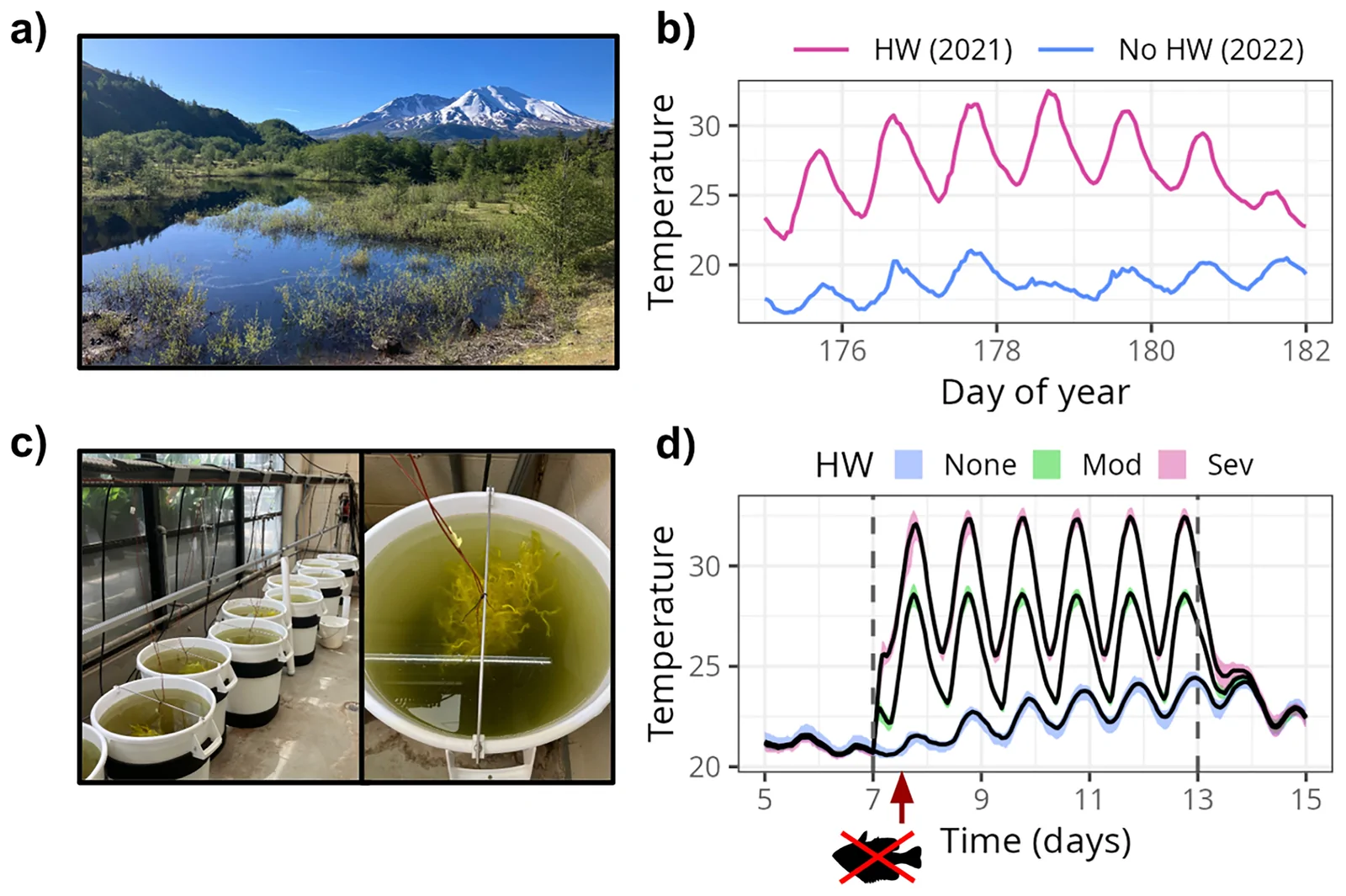
Capturing ecologically relevant signatures of heatwaves in a mesocosm experiment. (a) A photograph and (b) hourly temperature time series from a local pond ecosystem found in the hummocks of the Cascade Mountains near Mount St Helens (Pond H04, WA, USA) during the June 2021 Pacific Northwest heatwave (HW, pink line) and during typical June weather conditions in 2022 (No HW, blue line). (c) Photographs and (d) hourly surface temperature time series of the experimental mesocosms during heatwaves (see supplementary material, figure S1 for full time series). Coloured bands indicate the range of temperatures among replicate mesocosms within the no, moderate and severe heatwave (HW) treatments. Vertical dashed lines indicate the start and end of heating. Predator losses took place on the first day of the heatwave (red arrow).

Each mesocosm was prepared with a substrate layer, a leaf-litter bag, a temperature logger and an artificial plant refuge for zooplankton (details in supplementary material), then filled to a depth of 53 cm with tap water (approx. 75.5 l). To establish diverse, comparable plankton communities across the array, we inoculated stock mesocosms with phytoplankton sourced from Reed Lake and zooplankton from seven local ponds, manually removing conspicuous predator *Chaoborus* larvae prior to introducing zooplankton. We then introduced standardized aliquots of phytoplankton- and zooplankton-rich water to each experimental mesocosm, homogenizing the zooplankton community across the array beforehand to minimize initial compositional differences among mesocosms (full inoculation details in supplementary material).

After inoculation, we allowed 8 days for zooplankton to reproduce before introducing two zooplanktivorous pumpkinseed sunfish (*Lepomis gibbosus*) to each mesocosm. During pilot studies, we observed the active consumption of zooplankton by this fish species in glass aquaria, verifying that they have an important role as predators in this system exerting consumptive, top-down effects. Sunfish were captured from Reed Lake with minnow traps and kept in indoor aquariums for up to 15 weeks before being introduced into the mesocosms. We equalized total fish biomass across mesocosms by pairing one above and one below-average individual per tank (biomass range: 2.08–5.18 g; length range: 37.7–44.2 mm). Mesocosms were monitored daily. On five occasions when we observed a dead fish in a P+ mesocosm, we removed and replaced it immediately. To simulate predator mortality owing to heat stress, we captured all of the fish from the P− mesocosms on the first day of the heatwave using aquarium mesh fishnets, euthanized them by freezing for 48 hours and added two dead fish to each P− mesocosm. This decision was made to simulate predator mortality as an integrated event that includes both a reduction in top-down control and the release of nutrients from decomposing predator carcasses [9,12]. We additionally mixed the P+ mesocosms using fishnets in order to enact a similar disturbance on the first day of the heatwave.

### (b) Temperature and light control

The temperature control system for the heated mesocosm array is described in detail in [46]. We used this system to impose a week-long, temporally variable heatwave during the second week of the experiment (3–9 July). We recorded water temperature in each mesocosm with a HOBO Pendant temperature logger (Onset Computer Corporation; Bourne, MA, USA) located approximately 34 cm above the bottom of the container.

We designed the severe heatwave treatment to mimic water temperature measurements taken in a small pond typical of those found in the Cascade Mountain hummocks near Mount St Helens, WA: pond H04 (Washington, USA) during the June 2021 Pacific Northwest heatwave (figure 1a,b). This pond reached a maximum surface temperature of 33.4°C, far higher than the surface temperatures observed at this time in a typical year (figure 1b). To produce a similar level of temporal variation within the mesocosms, heater setpoints were determined via sinusoidal functions with a fixed daily temperature range of 7°C and maxima of either 32°C (severe heatwave) or 28°C (moderate heatwave) (figure 1d). Mesocosms in the no heatwave treatment remained at ambient temperature throughout the experiment (supplementary material, figure S1). To supplement incoming natural light to reach typical field levels, we lit the array with broad-spectrum, 5000K LED strips (Bridgelux; Fremont, CA, USA), suspended approximately 47 cm above the water surface, set to a daily 14:10 (on:off) schedule.

### (c) Sampling and data collection

Mesocosms were sampled twice weekly before and during the heatwave, then weekly after the heatwave. After gently homogenizing mesocosms, water was transferred to a bucket with an integrated depth sampler. A 200 ml homogenized whole-water sample was kept on ice for subsequent chlorophyll *a* analysis. Samples of small and large zooplankton were obtained by reverse filtration through two different sizes of mesh to ensure capturing a broad spectrum of zooplankton sizes: 750 ml were filtered through a 61 µm mesh to capture small-bodied zooplankton (Rotifera and nauplii), and a larger volume of 3 l was filtered through a 241 µm mesh to concentrate the comparatively larger crustacean zooplankton. Zooplankton samples were rinsed into separate 50 ml test tubes with tap water, preserved with Lugol solution and stored in the dark at 4**°**C until counting.

Zooplankton were enumerated with a stereomicroscope within 30 days of collection using a standardized subsampling protocol to estimate total counts for each taxon (full counting details in supplementary material). Zooplankton were identified to genus when possible before being aggregated into family-level functional groups (see below), with copepods identified to class and nauplii not identified further.

We estimated the biomass of each zooplankton group using a set of standard, published length–weight regressions [53–55]. After pooling zooplankton samples from all mesocosms on three sample dates (15 July, 29 July and 12 August), we measured 20 individuals from each of the common zooplankton groups and as many as feasible (minimum 7) from the six rarest groups; we then estimated the dry weight of each measured individual. Because only wet weight regressions were available for Cyprididae and Rotifera, we assumed dry-weight-to-wet-weight ratios of 0.44 and 0.1, respectively, for these taxa [56]. We multiplied the raw counts by the mean individual dry weight to estimate the total dry weight of each zooplankton group. For most analyses, we divided the zooplankton community into five key groups: Cyprididae, Rotifera, Cyclopidae, Chydoridae and Daphniidae, corresponding to major functional groups for fresh water consumers [57].

To measure total phytoplankton abundance, we performed non-acidified extractions of chlorophyll *a* in ethanol [58]. Fluorescence was quantified with a Trilogy fluorometer (Turner Designs; San Jose, CA, USA) using the non-acidified chlorophyll (CHL-NA) module and calibrated against commercial chlorophyll *a* standards (Turner Designs). We additionally collected whole-water samples on four dates (1 July, 9 July, 5 August and 19 August) and kept them frozen at −20**°**C for subsequent determination of total phosphorus (TP). To quantify TP, two replicates of each sample were reduced with ascorbic acid and molybdate, and absorbance was measured spectrophotometrically at 830 nm [59].

### (d) Statistical analysis

We used three statistical approaches to analyse the effects of heatwaves and predator losses on producer and consumer communities. First, we used generalized additive mixed models (GAMMs) fitted in R with the ‘mgcv’ package to test how each response variable collected over time (chlorophyll *a*, total zooplankton biomass, Daphniidae biomass, Rotifera biomass, Cyclopidae biomass, Chydoridae biomass, Daphniidae relative biomass, total phosphorus and Shannon diversity) responded to heatwave intensity (none, moderate and severe), fish (fish present versus absent) and their interaction over the course of the experiment. Similar to previous studies [9], Shannon diversity (*H*) was calculated as follows: $H\;=\;-\sum_{i\;=\;1}^{S}p_{i}\text{ln}\left(p_{i}\right)$, where *S* is the number of functional groups and $p_{i}$ is the relative biomass of each individual functional group. All models included parametric (fixed) effects for heatwave, fish and their interaction, along with a smoothing term for week fit separately for each unique combination of heatwave intensity and fish treatment using thin-plate regression splines. This structure allowed the temporal trajectory of each response variable to differ nonlinearly among treatment combinations. To account for the nonindependence of repeated measurements from the same mesocosm over time, all models included a mesocosm-level random effect, specified as a random-effect smooth (bs = ‘re’). For each response variable, we fit six candidate GAMMs that differed in distribution (Gaussian or negative binomial) and level of complexity (parametric terms only, parametric terms and fixed smooth term, or parametric terms and both fixed and random smooth terms). We then used Akaike information criterion (AIC) to select the best model and optimize the basis dimension (*k*, ranging from *k* = 3 to *k* = 8) of the smooth term over the range of possible values. To assess potential confounding effects of row position owing to differences in light availability between rows, we compared each best-selected GAMM against an equivalent model containing row as an additional fixed parametric term using AIC; yet, row was not retained in any GAMM model (all ΔAIC < 2). All models were fit via restricted maximum likelihood (REML).

For most response variables (chlorophyll *a*, total zooplankton biomass and biomass of Rotifera, Cyclopidae and Chydoridae), the best model took the form of including a random slope that allowed the temporal trend across weeks to vary among mesocosms. For Daphniidae biomass, Shannon diversity and total phosphorus, a simpler random effect intercept for mesocosm was used instead. The final form of each GAMM is listed in table S1 in the supplementary material.

Second, we assessed the effects of heatwave intensity, predator loss and their interaction on two days—the first sampling day after the heatwave ended (15 July) and the final day of the experiment (19 August)—using two-way ANOVA. We additionally used two-way ANOVA to assess whether water temperatures differed among treatments. We log-transformed all chlorophyll and zooplankton data by adding a value of one and then taking the logarithm. Similarly, we used two-way ANOVA to test for treatment differences in three consumer community properties that were calculated across time: (i) community stability, calculated as the temporal mean of zooplankton biomass divided by the temporal standard deviation of zooplankton biomass [60], such that high values indicate greater stability, (ii) community synchrony, calculated as the variance of aggregated zooplankton functional group biomass values divided by the squared sum of the standard deviations of each individual functional group biomass time series [61], such that values of 0 indicate perfect asynchrony and values of 1 indicate perfect synchrony, and (iii) community trajectory metrics (described below) [62]. We applied the previously described procedure to evaluate whether to include row as a covariate: row was retained as a fixed covariate in two models where ΔAIC > 2 (mean mesocosm temperature and August 19 chlorophyll *a*), though its inclusion did not alter the significance or direction of treatment effects in either case. We confirmed that ANOVA models met the assumptions of homogeneity of variance and normality.

Third, to analyse changes in zooplankton abundance and community composition through time, we performed community trajectory analysis (CTA) [62]. CTA allowed us to quantify how the community collectively responded through time to the experimental treatments. We generated a matrix of Bray–Curtis dissimilarities between samples based on the biomass of zooplankton groups after applying a Wisconsin double standardization. We then used the ‘ecotraj’ R package to generate community trajectories and calculate several metrics related to the trajectories [62]. These metrics were as follows: the path length, defined as the sum of the lengths of each individual trajectory segment; the mean of the angles between adjacent path segments; and the directionality of the trajectory. In addition, we assessed the difference between each pairwise treatment combination by calculating the mean of the Bray–Curtis dissimilarities between the mesocosm communities across all sampling dates.

For ANOVA and GAMM analyses, we considered treatment effects to be statistically significant based on *α* = 0.05. For response variables where time series existed, we considered instances in which treatments exhibited non-overlapping 95% confidence intervals to be statistically significantly different from one another. Prior to initiating experimental treatments, we observed no evidence of meaningful differences in experimental conditions. Additionally, initial mean fish biomass did not differ across experimental treatments (*F*_2,9_ = 0.22, *p* = 0.81). All analyses were conducted in R v.4.4.1 .

## 3. Results

### (a) Experimental conditions

The mesocosm array allowed producing heatwaves that mimicked the conditions of a recent extreme temperature event in Western North America (figure 1). During the week-long heatwave phase of the experiment, the mean mesocosm temperature significantly differed between all three heatwave intensity groups (figure 1d; ANOVA; *F*_2,17_ = 1410, *p* < 0.001, Tukey HSD post hoc) but not between predator present (P+) and predator loss (P− treatments; *F*_1,17_ = 1.86, *p* = 0.191). The mean temperature in the ‘moderate heatwave’ mesocosms was 3.5°C warmer than the ‘no heatwave’ mesocosms, while the mean temperature in the ‘severe heatwave’ mesocosms was 6.6°C warmer than the ‘no heatwave’ mesocosms (figure 1d). The daily minima and maxima of the severe heatwave mesocosms were similar to the temperatures experienced in a local reference pond (pond H04) during the 2021 Western North American summer heatwave (figure 1b). Mean mesocosm temperature before and after the heatwave did not differ by heatwave intensity nor by predator loss (supplementary material, figure S1).

### (b) Producer and consumer biomass responses

Primary producer and total zooplankton biomass exhibited stronger responses to predator loss than to heatwave intensity (figure 2a–c). Chlorophyll *a* tended to increase gradually over time in P+ mesocosms while remaining relatively stable in P- mesocosms. While chlorophyll *a* was 2.5-fold greater in P+ mesocosms than P− mesocosms at the end of the experiment (figure 2a; two-way ANOVA predator loss; *F*_1,18_ = 10.7, *p* = 0.004), the effect of predator loss was not significant over the full time series (Wald test; *χ*^2^_1_ = 0.870, *p* = 0.351) or in the immediate aftermath of the heatwave (two-way ANOVA predator loss; *F*_1,18_ = 0.19, *p* = 0.67). The heatwave treatments produced an immediate increase in chlorophyll *a* (figure 2a; two-way ANOVA heatwave intensity, F_2,18_ = 13.98, *p* <0.001), such that total chlorophyll was 2- and 3-fold elevated in moderate and severe heatwave treatments, respectively, relative to the no heatwave treatment. However, neither heatwave intensity (Wald test; *χ*^2^_2_ = 3.49, *p* = 0.174) nor its interaction with predator loss (Wald test; *χ*^2^_2_ = 2.41, *p* = 0.300) significantly affected chlorophyll *a* over the full time series.

**Figure 2.**
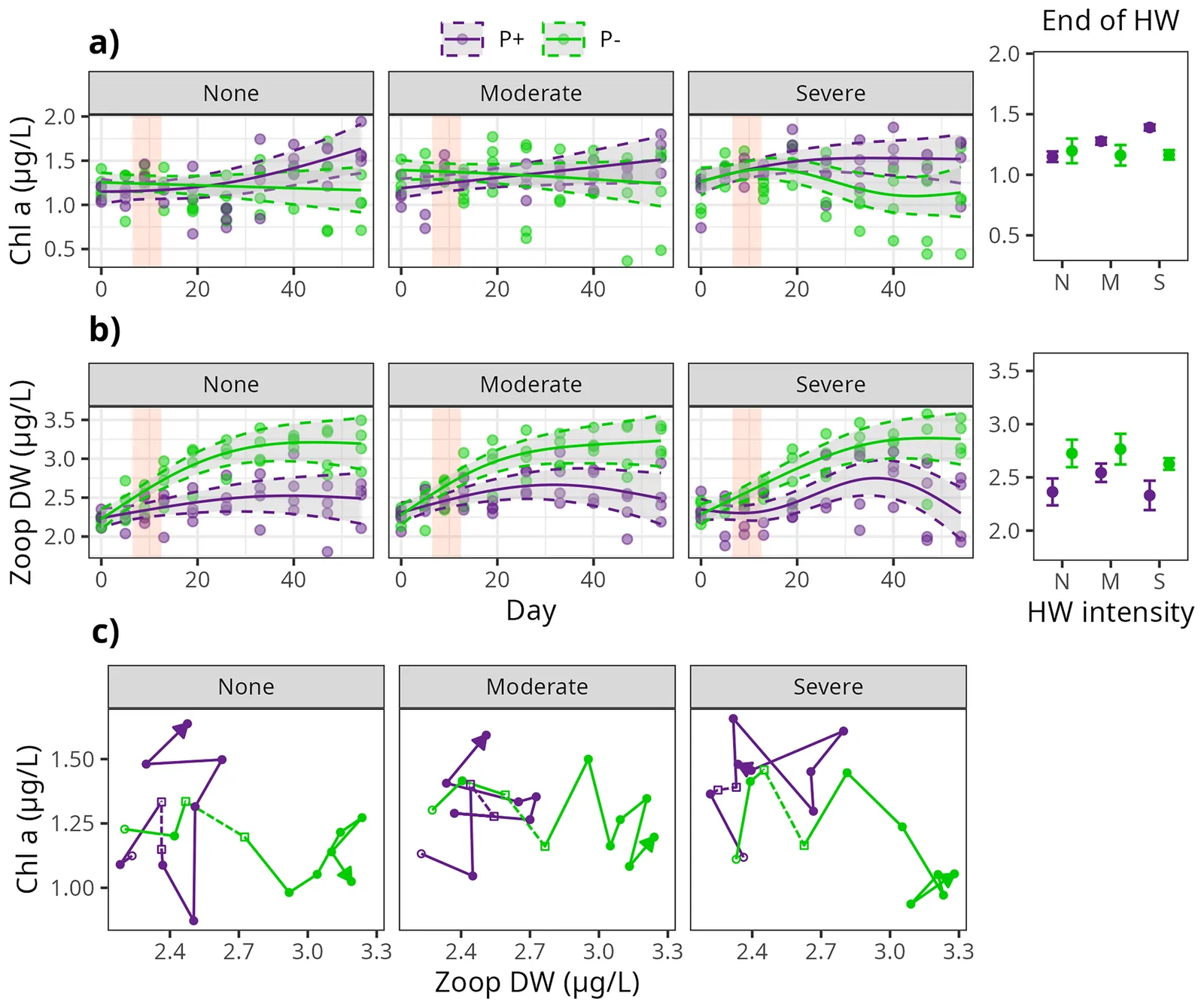
Producers and consumers are more strongly affected by predator loss than by heatwaves. Time series show (a) log-transformed chlorophyll *a* and (b) log-transformed total zooplankton dry biomass in no (N), moderate (M) and severe (S) heatwave conditions with (purple, P+) and without (green P−) fish predators persisting. Data from individual mesocosms (small points) are plotted along with generalized additive mixed model fits, where solid lines indicate model predictions and shaded regions between dashed lines indicate 95% confidence intervals. Red shading illustrates the heatwave period. Rightmost panels compare treatments (mean +/− SE) at the end of the heatwave (day 19). (c) Coupled time series show relationships between mean chlorophyll *a* and zooplankton dry biomass over time among treatments, where open circles represent the first sampling date, open squares and dashed lines indicate the heatwave period, and arrows indicate the final sampling date.

The primary impact of predator loss was to increase zooplankton biomass, such that zooplankton biomass increased over time in P− mesocosms yet remained relatively stable in P+ mesocosms (figure 2b). Total zooplankton biomass was 3-fold higher in P− than P+ mesocosms over the full time series (Wald test; *χ*^2^_1_ = 18.6, *p* < 0.001). These treatment effects diverged during the heatwave such that differences existed immediately after the heatwave (two-way ANOVA predator loss; *F*_1,18_ = 32.91, *p* < 0.001) and became more prominent on the final day of the experiment (figure 2b; two-way ANOVA predator loss, *F*_1,18_ = 44.6, *p* < 0.001). Immediately following the heatwave period, zooplankton biomass did not differ across heatwave treatments (two-way ANOVA heatwave intensity; *F*_2,18_ = 0.40, *p* = 0.67), and there were no persistent effects of heatwave intensity on total zooplankton biomass over the full time series (Wald test; *χ*^2^_2_ = 1.05, *p* = 0.593) or on the final day of the experiment (two-way ANOVA heat intensity; *F*_2,18_ = 1.14, *p* = 0.341).

Collectively, producer biomass increased to a large extent, while zooplankton biomass increased only modestly in mesocosms where predators persisted (figure 2c). By contrast, mesocosms experiencing die-offs resulted in substantial zooplankton proliferation yet declines in standing producer biomass (figure 2c). The effects of heatwaves for aggregate zooplankton and phytoplankton communities were subtle, being most evident in the immediate aftermath of the heatwave for increasing producer biomass (figure 2c).

### (c) Population- and community-level responses

The loss of predators, and to a lesser extent heatwave intensity, altered the population dynamics of individual zooplankton taxa (supplementary material, figure S3). Over the full time series, P- mesocosms had significantly higher biomass of large-bodied zooplankton taxa compared to P+ mesocosms: 23-fold higher Daphniidae biomass (figure 3a; supplementary material, figure S3a; Wald test; *χ*^2^_1_ = 15.0, *p* < 0.001) and 2-fold higher Cyclopidae biomass (supplementary material, figure S3b; *χ*^2^_1_ = 8.42, *p* = 0.004), but only 41% more biomass of medium-sized Chydoridae, although this was not statistically significant (figure 3a; supplementary material, figure S3c; *χ*^2^_1_ = 3.11, *p* = 0.078). Conversely, P− mesocosms had significantly less rotifer biomass (the smallest zooplankton we enumerated) than P+ mesocosms (a 49% reduction, figure 3a; supplementary material, figure S3d; Wald test; *χ*^2^_1_ = 24.2, *p* < 0.001). A significant interaction between heatwave intensity and predator loss was present for Daphniidae (Wald test; *χ*^2^_2_ = 8.97, *p* = 0.011), as the severe heatwave led to a 57% reduction in Daphniidae biomass among P+ mesocosms but a 24% increase among P− mesocosms. The interaction between heatwave intensity and predator loss had a significant effect on Rotifera as well (supplementary material, figure S3d; Wald test; *χ*^2^_2_ = 8.25, *p* = 0.016). The moderate heatwave led to a 2-fold increase in rotifer biomass among P− mesocosms but a 24% decrease among P+ mesocosms, while the severe heatwave exerted more modest effects (figure 3a).

**Figure 3.**
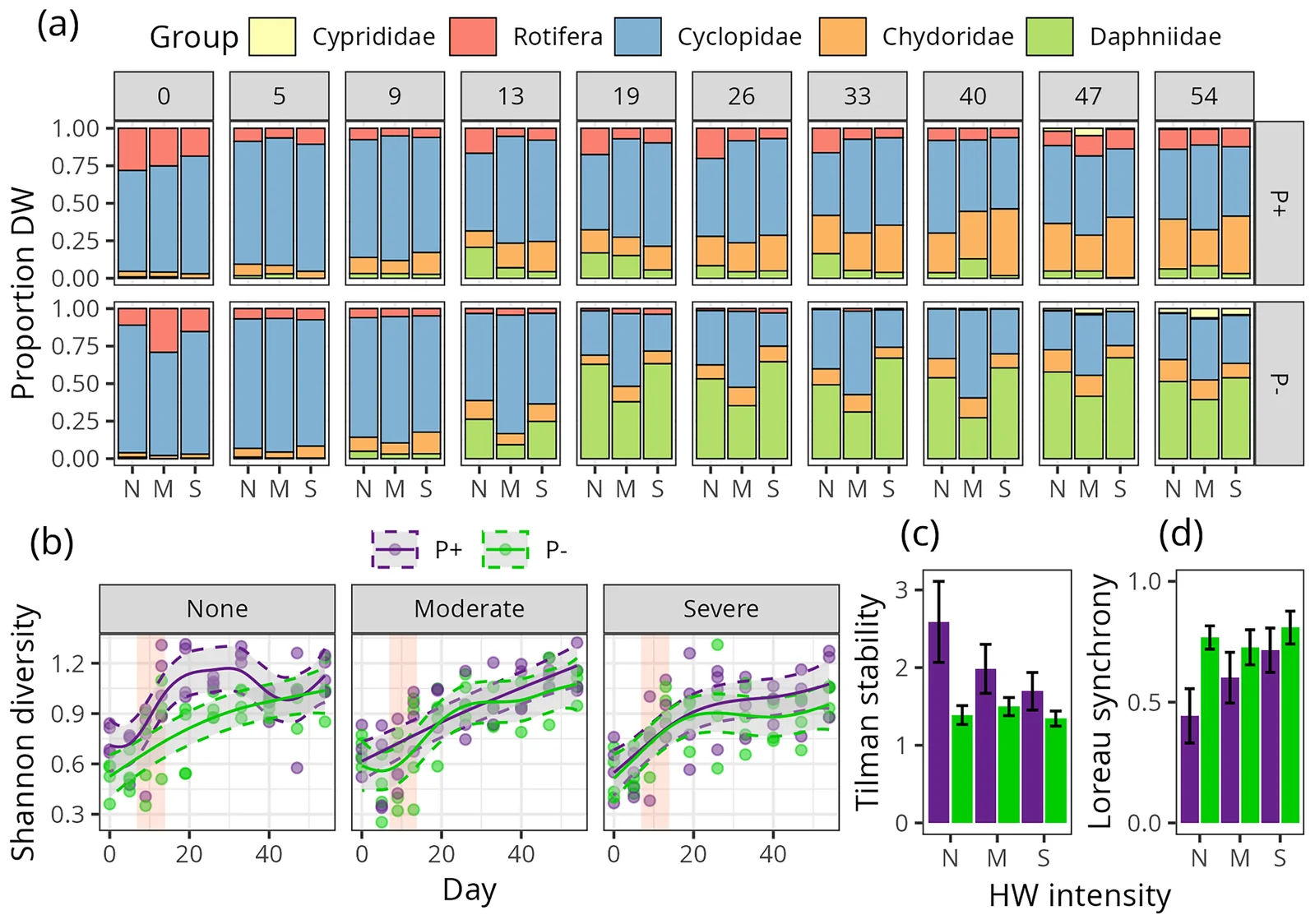
Heatwaves and predator loss affect the diversity, stability and synchrony of the zooplankton community. (a) The proportion of the total zooplankton dry weight belonging to each of five major taxonomic groups (see ‘Methods’) through time in no (N), moderate (M) and severe (S) heatwave conditions with (P+, purple) and without (P−, green) fish predators persisting. Facets are arranged chronologically from left to right, with titles indicating the sampling day; Rotifera and Chydoridae had small average body sizes, and Daphniidae had the largest. (b) The Shannon diversity index of each sample calculated based on the biomass values of five major groups of zooplankton, with the heatwave period shown in red shading. (c) Tilman temporal stability and (d) Loreau synchrony indices are summarized by treatment group, where bars indicate the mean values and error bars are +/− 1 s.e.

Predator loss also strongly affected the composition and temporal variability of the zooplankton communities, while heatwave intensity modified community composition to a lesser extent (figure 3a). The initial communities were dominated by Cyclopidae (76%) and rotifer biomass (21%) but contained little cladoceran biomass. During the post-heatwave phase of the experiment, Cyclopidae remained the dominant group in P+ mesocosms (56%), with substantial contributions from Chydoridae and Rotifera (27 and 10%, respectively; figure 3a). By contrast, Daphniidae constituted the bulk of the biomass in P− mesocosms after the heatwave (49%), while Cyclopidae, Chydoridae and Rotifera represented lower portions (33, 8 and 3%, respectively). Among P− mesocosms, the moderate heatwave significantly reduced the relative biomass of Daphniidae following the heatwave until day 40 to approximately 25% of consumer biomass, compared to approximately 50% in both the no heatwave and severe heatwave groups (figure 3; supplementary material, figure S4).

While Shannon diversity tended to increase over time, P− mesocosms had approximately 10% lower Shannon diversity, on average, than P+ mesocosms (figure 3b; Wald test; *F*_1,202.6_ = 7.75, *p* = 0.006). Across the different heatwave treatments, predator loss led to the greatest reductions in Shannon diversity (−18%; figure 3b) for mesocosms not receiving a heatwave; this significant reduction in Shannon diversity emerged during the predator loss and persisted until day 32. P− mesocosms had significantly lower community stability (figure 3c; two-way ANOVA predator loss; *F*_1,18_ = 8.89, *p* = 0.008) and higher synchrony (two-way ANOVA predator loss; *F*_1,18_ = 6.69, *p* = 0.019) than P+ mesocosms (figure 3d). Heatwave intensity did not impact stability or synchrony (figure 3c; two-way ANOVA heatwave intensity on stability: *F*_2,18_ = 1.36, *p* = 0.28; figure 3d, two-way ANOVA heatwave intensity on synchrony: *F*_2,18_ = 1.62, *p* = 0.23); the interactive effects were not statistically significant for stability (two-way ANOVA predator loss × heatwave intensity: *F*_2,18_ = 1.34, *p* = 0.288) or synchrony (two-way ANOVA predator loss × heatwave intensity, *F*_2,18_ = 1.06, *p* = 0.368).

### (d) Temporal trajectory of communities

Predator loss caused zooplankton communities to follow substantially different trajectories during and after the heatwaves (figure 4a). Trajectories were similar across treatments before the heatwave, with downward movement along Axis 2 indicating declines in Rotifera and increases in Cyclopidae (figure 4b). During the heatwave, however, P+ and P− mesocosms sharply diverged. P− mesocosms displayed substantial movement along Axis 1, driven largely by increases in Daphniidae biomass, while trajectories of P+ mesocosms were more constrained and tended to converge towards the initial community state near the end of the time series. Indeed, trajectories from P− mesocosms had 36% greater directionality than P+ mesocosms (two-way ANOVA predator loss; *F*_1,18_ = 19.5, *p* < 0.001), indicating a consistent shift in zooplankton community composition over time. By contrast, neither heatwave intensity nor its interaction with predator loss significantly impacted directionality (two-way ANOVA heatwave intensity; *F*_2,18_ = 0.257, *p* = 0.776; two-way ANOVA heatwave intensity × predator loss; *F*_2,18_ = 1.415, *p* = 0.269). P− mesocosms additionally had significantly lower (a 23% reduction compared to P+) mean trajectory angles (figure 4c; two-way ANOVA predator loss; *F*_1,18_ = 11.8, *p* = 0.003), indicating less abrupt changes in the zooplankton community through time. Heatwave intensity did not significantly impact trajectory angles (figure 4c; two-way ANOVA heatwave intensity; *F*_2,18_ = 2.66, *p* = 0.097). Neither predator loss, heatwave intensity nor their interactions had a significant effect on trajectory path length (two-way ANOVA predator loss; *F*_1,18_ = 0.749, *p* = 0.398; two-way ANOVA heatwave intensity; *F*_2,18_ = 0.439, *p* = 0.652; two-way ANOVA heatwave intensity × predator loss; *F*_2,18_ = 0.221, *p* = 0.804) indicating that communities exhibited similar magnitudes of change through time.

**Figure 4.**
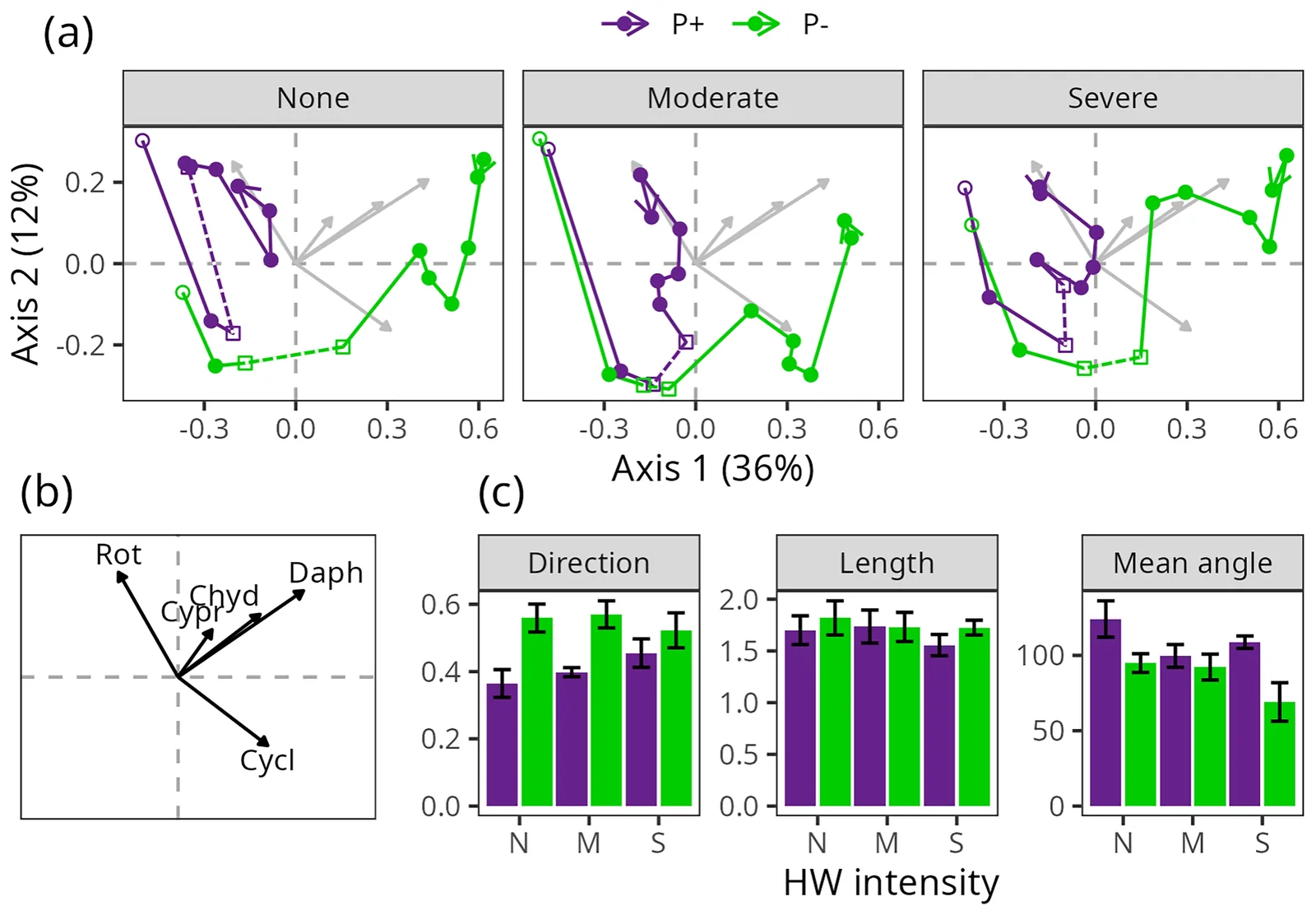
Predator loss and heatwaves have lasting effects on the temporal trajectories of zooplankton communities. (a) Mean trajectory paths over 54 days are shown for no, moderate and severe heatwave conditions with (P+, purple) and without (P−, green) fish predators persisting, with arrows indicating the ends of the trajectories and open squares and dashed lines indicating the heatwave. Trajectories were generated by performing principal coordinate analysis on Bray–Curtis dissimilarities across treatments and across time, using biomass data from the five major zooplankton groups. The light grey vectors in (a) indicate the direction of the association between each zooplankton group and the principal axes as shown in panel (b), and the lengths of the vectors are scaled to the strength of the correlation (Pearson *r*). (c) The directionality, path length and mean angle of the trajectories are summarized by treatment, where coloured bars indicate the mean values and error bars are +/− 1 s.e.

Zooplankton communities from environments lacking predators tended to exhibit relatively large differences in community composition relative to those containing predators (Bray–Curtis dissimilarity > 0.45), while communities from the same predator loss treatment were generally similar in composition regardless of their respective heatwave treatments (supplementary material, figure S5).

### (e) Nutrient response

Total phosphorus (TP) was 55% higher in P− than P+ mesocosms over the full time series (Wald test; *F*_1,66.5_ = 14.0, *p* < 0.001; supplementary material, figure S6) and 135% higher on the final day of the experiment (ANOVA; *F*_1,18_ = 16.9, *p* < 0.001), but the magnitude of these effects differed by heatwave treatment. Heatwaves dampened the positive effect of predator loss on TP, as evidenced by the significant interactive effect (Wald test; *F*_2,66.5_ = 3.47, *p* = 0.037). By the end of the experiment, predator loss increased mean TP by only 6% among severe heatwave mesocosms, compared to a 370% increase among no heatwave mesocosms.

## 4. Discussion

Our findings demonstrate that the persistence of predators during periods of elevated temperatures is the critical determinant of how fresh water food webs respond during heatwaves. Although all treatments experiencing heatwaves exhibited ecological change, the most consequential ecological shifts arose only when predator loss occurred during the onset of heatwaves. As such, predator loss was the main driver of change throughout the experiment, irrespective of heatwave intensity. This finding is critical because heatwaves are emerging as one of the most destabilizing facets of global change [4,7,22] yet are typically viewed through the lens of producing severe, direct, thermal stress rather than altered trophic interactions precipitated by predator loss.

By the end of the experiment, predator loss altered expected ecological dynamics irrespective of heatwave severity through a classic trophic cascade: consumer biomass increased, primary producer biomass declined, and nutrient concentrations rose [15,63,64]. Zooplankton assemblages shifted from small- to large-bodied taxa, consistent with the disappearance of size-selective predation [65,66]. Communities lacking predators were less diverse, less temporally stable and more synchronized, all patterns expected when dominant competitors are no longer selectively suppressed by predators [20,67]. Such patterns mirror changes observed following natural, heat-induced fish die-offs in lake systems [68], yet our experiment shows mechanistically that predator loss—not the direct thermal effects of heatwaves—generates both the immediate and persistent restructuring of producer and consumer dynamics.

By contrast, the direct effects of temperature were comparatively subtle and strongly contingent upon predator persistence. When predators survived, heatwaves propagated through the food web via increased top-down control, consistent with metabolic-theory predictions [26] and warming experiments showing elevated predation rates at higher temperatures [69,70]. Increased consumption of large-bodied zooplankton explains the reductions in these taxa and is consistent with keystone predator theory predicting declines in prey evenness under intense predation [71]. The only main effect of temperature consistent across predator treatments was a short-lived increase in chlorophyll immediately after heatwaves, probably arising from producer–consumer stoichiometric shifts [72] and the higher temperature sensitivity of photosynthesis relative to nutrient uptake [73,74]. Thus, the effects of temperature extremes alone varied considerably among trophic levels.

The greatest temperature-associated compositional shifts occurred when predators were lost. This finding aligns with work showing amplified heatwave impacts in systems lacking predators [75], yet the responses we observed were distinctly nonlinear. For example, large-bodied Daphniidae, which are often sensitive to warming [76], performed poorly at intermediate heatwaves but were abundant under control and severe heatwaves, whereas cyclopoids showed the opposite pattern. These results support evidence that heatwaves may impose limited stress on zooplankton in the absence of predators [77], probably owing to the ability of many zooplankton to tolerate high temperatures [78] and nightly thermal relief that allows for some degree of physiological repair [79]. Importantly, thermally driven fish die-offs often occur at water temperatures well below their lethal temperatures [9], suggesting that predator mortality during heatwaves often reflects interacting physiological constraints, such as oxygen limitation, rather than simple upper thermal limits. Under these circumstances, direct heatwave effects on zooplankton may be more diminished in nature than the relatively severe heatwaves imposed in our experiment.

Our study both complements and expands on previous work characterizing ecological responses to altered thermal environments. Previous predator-manipulation heatwave experiments typically manipulate predator densities well in advance of the heatwave, rather than being imposed during the heatwave [35–38]. This distinction is critical. Communities that lose predators via *in situ* mortality experience transient dynamics, nutrient inputs from decomposing predator carrion [9,12,44,47] and sudden shifts in food web structure that cannot be captured by comparing predator-present versus predator-absent communities established prior to warming [9]. Because predators are often the trophic group most sensitive to temperature perturbations [80], heatwave-driven predator loss may broadly represent the dominant mechanism through which thermal extremes acutely reorganize natural communities. Consequently, communities with heat-tolerant predators are probably more resilient to thermal extremes than those in which predators perish and offer a forecast for how communities may respond to climatic extremes [6].

Interpreting our results in the context of natural communities requires careful consideration of both the strengths and limitations of mesocosm approaches and the ways our findings may depart from those in other ecosystems [12]. Mesocosms necessarily simplify natural systems, capturing less biodiversity and spatial complexity than natural systems [81,82]. However, this simplification is also a key strength since it allows for the level of experimental control and replication needed to isolate causal effects of extreme warming—something that is logistically infeasible and ethically dubious in natural lakes, where heatwaves and predator loss cannot be replicated or manipulated at scale. Our mesocosms were designed to resemble the depth and composition of the small ponds from which our communities were derived, systems that are globally abundant [83], highly thermally variable [84] and particularly vulnerable to high-magnitude heatwaves [4,45]. As zooplankton densities increased during the first weeks of this experiment, these communities may most closely resemble late spring to early summer communities. Because the water was stationary, our results may not directly inform responses in lotic ecosystems where flow dynamics, hydrological conductivity and dispersal processes may alter the post-heatwave community response. Other important differences remain from natural lentic ecosystems. For example, in larger and deeper lakes, fish may seek thermal refuges not present in our mesocosms [18]. Additionally, many fish kills are partial rather than complete [6], which could dampen, but not eliminate, post-heatwave trophic cascades. As such, the ecological effects observed in P− treatments may represent an upper bound of post-heatwave trophic disruptions. Finally, while we attribute predation as the primary mode by which fish predators impacted zooplankton in the mesocosms, fish may also influence zooplankton via non-consumptive effects [85].

Although ecologists have long recognized the central roles of predators [13,14] and temperature [23,26,27] in shaping ecosystems, contemporary climate extremes increasingly couple these two forces by reshaping predator communities themselves [6,48]. Our results show that predator extirpation arising during heatwaves, which diminish top-down control and provide a carcass-derived nutrient subsidy [9], is probably the primary mechanism driving ecological change in fresh water food webs, producing far larger and more persistent changes than direct thermal stress on lower trophic levels. As heatwaves intensify across the globe, affecting both fresh water [6,48] and terrestrial [86] environments alike, identifying the conditions that enable predator persistence may prove critical for maintaining ecosystem stability.

## Supplementary Material



## Data Availability

All data for analyses are available at a permanent Zenodo repository [87]. Supplementary material is available online [88].
