# Supplementary material for: Predator loss amplifies the disruptive impacts of heatwaves
Source: Proc Biol Sci. 2026 Nov 4;293(2082):20262081. doi: 10.1098/rspb.2026.2081 (PMC13730808; doi:10.1098/rspb.2026.2081)
Supplement: Supplementary file 1 [file rspb.2026.2081.zip › rspb.2026.2081/rspb20262081_si_001.docx]

**Predator loss amplifies the disruptive impacts of heatwaves**

This PDF file includes:

- Supporting text (Supplemental Methods)
- Figures S1 to S6
- Table S1

**Supporting Text:**

Mesocosm construction details: We first covered the bottom of each mesocosm with a thin layer of aquarium gravel. Then, we added a small laundry bag containing: 10 g of dry leaves (>90% of which came from bigleaf maple, *Acer macrophyllum*), as a source of dissolved organic carbon and microbial inoculum; a data logger set to take hourly records of water temperature; and a handful of aquarium gravel to sink the bag. Mesocosms were filled to a depth of 53 cm with tap water (~75.5 L). Each mesocosm also received one 45-cm tall, artificial plant constructed of polypropylene rope to provide a predation refuge for zooplankton.

To promote the establishment of a diverse, local plankton community, we first inoculated two outdoor 75.5-L containers (hereafter referred to as the stock mesocosms) with phyto- and zooplankton sourced from multiple local ponds. We set up the stock mesocosms according to the above steps and covered them with 1-mm fiberglass mesh to prevent establishment by terrestrial insects. Two days after filling, we inoculated them with 4.8 L of 250-µm filtered water from Reed Lake (Oregon, USA) to introduce phytoplankton, as this amount of lake water had previously been shown to provide adequate food to freshwater zooplankton. Over the next two weeks, we collected concentrated zooplankton samples by filtration through a 61 µm mesh net from 7 natural ponds located in northwestern Oregon and southwestern Washington, USA. The concentration of collected zooplankton in these samples was not recorded. After manually removing conspicuous *Chaoborus* larvae (a predator of zooplankton), we introduced the zooplankton to the stock mesocosms. *Chaoborus* were detected in ~1% (3 of 240) of sampling events during the experiment, indicating that they were introduced and persisted at very low densities.

Two weeks after establishing the experimental mesocosms, we filtered water from Reed Lake, OR, USA through a 250 µm mesh (to exclude large grazers) and inoculated each experimental mesocosm with 2.5 L of the water sample in order to add an initial phytoplankton community, consistent with ratios used in previous studies [53]. Three days later, we inoculated each experimental mesocosm with 2.2 L of well-mixed water from each of the two zooplankton stock mesocosms (4.4 L total). We homogenized the initial zooplankton community across the array by removing 2 L of water from each mesocosm, pooling these samples into a single container, and then adding back 2 L of the combined and homogenized water.

Zooplankton counting details: Zooplankton were enumerated with a stereo microscope (Nikon Instruments; Melville, NY, USA) within 30 days of collection. Due to a dramatic increase in the density of copepods and cladocerans over the course of the experiment (Fig. S3a,b), we only counted 40% of the total volume (equivalent to 1.2 L of mesocosm water) of large-bodied zooplankton samples collected on and after July 9, unless 50 or fewer total individuals of all species were encountered in this volume, in which case we proceeded to count the entirety of the sample. For all small zooplankton samples, we counted a 1 mL subset in a Sedgwick Rafter chamber and then multiplied by the total volume of the sample to estimate the total count. We counted each taxon until we reached the end of the slide or reached a count of 100 individuals; in the latter case, we divided by the counted proportion of the slide to estimate the total count for that taxon. Zooplankton were identified to genus when possible before being aggregated to families (see main text), although mature copepods were identified to class and copepod nauplii were not identified further.

**Supplementary Figures:**

**Figure S1**. Temperature logger data collected hourly throughout the experiment and displayed by heatwave treatment, as displayed in Fig. 1d. The three levels of heatwave intensity were None, Moderate (Mod), and Severe (Sev). Heatwaves occurred between the vertical dashed lines.

**Figure S2.** Schematic to show the assignment of mesocosms to predator and heatwave treatments. Treatments were blocked by row and randomly assigned in each row. Each mesocosm is represented by a blue circle. Heatwave treatments are indicated as no heatwave (N), moderate heatwave (M), or severe heatwave (S). Fish conditions are indicated as predators removed during heatwave (P-) vs predators persisting (P+).

**Figure S3.** Responses of key zooplankton taxa biomass to predator loss and heatwaves, where the y-axis measures log-transformed dry weight (µg/L). Data from individual samples (circles) are plotted along with generalized additive mixed model fits for each variable, where solid lines indicate mean model predictions and shaded regions between dashed lines indicate 95 % confidence intervals. Biomass time series in no (N), moderate (M), and severe (S) heatwave conditions with (purple, P+) and without (green P-) fish predators persisting are shown for Daphniidae (a), Cyclopidae (b), Chydoridae (c), and Rotifera (d). Red shading illustrates the heatwave period. Rightmost panels compare heatwave and predator loss treatments (mean +/- SE) immediately after the heatwave (Day 19).

**Figure S4.** Relative biomass of Daphniidae throughout the experiment, with GAMM (solid lines are mean model predictions, dashed lines are 95% confidence intervals) fits by heatwave and predator (predator present: P+, predator absent: P-) treatment group. Red bars above the plot indicate days when a significant difference between heatwave treatments was detected, inferred from a lack of overlap between GAMM 95% confidence intervals.

**Figure S5.** Mean pairwise Bray-Curtis dissimilarities in zooplankton communities between treatment groups, predator levels: no predator (P-), predator present (P+); heatwave levels: none (N), moderate (M), severe (S). The mean Bray-Curtis index across the 10 sampling dates was calculated for each pair of distinct mesocosms, and these values were then averaged by treatment combination (*n* = 6 for cells on the diagonal, and *n* = 16 for all other cells).

**Figure S6.** Responses of total phosphorus over time to predator loss and heatwaves. Data from individual samples on 4 dates are plotted along with a generalized additive mixed model fit, where solid lines indicate mean model predictions and shaded regions between dashed lines indicate 95 percent confidence intervals. Red shading illustrates the heatwave period.

**Table S1**. Final GAMM formulas (presented as R scripts) for the response variables, using the “mgcv” package in R. Models include parametric (fixed) effects for heatwave severity (hw; none, moderate, severe), fish status (fish; removed vs. remaining during heatwave), and their interaction, together with a smooth term for day fit separately for each unique combination of heatwave intensity and fish treatment (via by=interaction(hw, fish), using thin-plate regression splines (bs="tp")). All models include a mesocosm-level random effect, specified as a random effect smooth (bs=“re”). The basis dimension (k) for these smooths was chosen independently for each response variable, and error distributions were selected between negative binomial (family = “nb”) or Gaussian (family = “gaussian”). A negative binomial distribution was selected for chlorophyll *a*, total zooplankton biomass, and taxon-specific biomass values, while a Gaussian distribution was selected for Shannon diversity, total phosphorus, and relative Daphniidae biomass. All models were fit via restricted maximum likelihood (REML) with rows containing missing data excluded (na.action=na.omit). As one example, the equation for the Chlorophyll *a* response variable is:

$\log\left( \mu_{ij} \right) = \beta_{0} + \beta_{1}{hw}_{j} + \beta_{2}{fish}_{j} + \beta_{3}\left( {hw}_{j} \times{fish}_{j} \right) + f_{{hw}_{j},{fish}_{j}}\left( {day}_{i} \right) + b_{j} \cdot{day}_{i}$ .

Here, $\mu_{ij}$ is the expected value for Chlorophyll *a* based on time point *i* in mesocosm *j.* $\beta_{0}$ is the intercept; $\beta_{1},\beta_{2},\mathrm{and} \beta_{3}$ are fixed effects of heatwave (hw), fish treatment, and their interaction; hwⱼ and fishⱼ are the categorical heatwave intensity (3 levels) and fish treatment (2 levels) factors; $f_{{hw}_{j},{fish}_{j}}\left( {day}_{i} \right)$ is a thin-plate regression spline smooth of day (basis dimension k = 4), fitted separately for each unique heatwave × fish combination; bⱼ is a mesocosm-specific random slope for day, accounting for repeated measures within mesocosms over time.

| Response Variable | Figure | GAMM formula |
| --- | --- | --- |
| Chlorophyll *a* | Fig. 2 | gam(Total_chl ~ hw*fish+s(day, by=interaction(hw, fish), bs="tp", k=4) + s(day, mesocosm, bs="re"), family="nb", method="REML", na.action=na.omit) |
| Total zooplankton biomass | Fig. 2 | gam(All_zoop ~ hw*fish+s(day, by=interaction(hw, fish), bs="tp", k=4) + s(day, mesocosm, bs="re"), family="nb", method="REML", na.action=na.omit) |
| Daphniidae biomass | Fig. S3 | gam(Daph_tot ~ hw*fish+s(day, by=interaction(hw, fish), bs="tp", k=4) + s(mesocosm, bs="re"), family="nb", method="REML", na.action=na.omit) |
| Rotifera biomass | Fig. S3 | gam(Rot_tot ~ hw*fish+s(day, by=interaction(hw, fish), bs="tp", k=5) + s(day, mesocosm, bs="re"), family="nb", method="REML", na.action=na.omit) |
| Shannon diversity | Fig. 3b | gam(Shannon ~ hw*fish+s(day, by=interaction(hw, fish), bs="tp", k=8) + s(mesocosm, bs="re"), family="gaussian", method="REML", na.action=na.omit) |
| Total phosphorus | Fig. S6 | gam(Total_phos ~ hw*fish+s(day, by=interaction(hw, fish), bs="tp", k=3) + s(mesocosm, bs="re"), family="gaussian", method="REML", na.action=na.omit) |
| Cyclopidae biomass | Fig. S3 | gam(Cycl_tot ~ hw*fish+s(day, by=interaction(hw, fish), bs="tp", k=6) + s(day, mesocosm, bs="re"), family="nb", method="REML", na.action=na.omit) |
| Chydoridae biomass | Fig. S3 | gam(Chyd_tot ~ hw*fish+s(day, by=interaction(hw, fish), bs="tp", k=4) + s(day, mesocosm, bs="re"), family="nb", method="REML", na.action=na.omit) |
| Daphniidae relative biomass | Fig. S4 | gam(Daph_tot ~ hw*fish+s(day, by=interaction(hw, fish), bs="tp", k=6) +s(day, mesocosm, bs="re"), family="gaussian", method="REML", data=b_rel, na.action=na.omit) |
